# Shoulder joint angles in supine and upright imaging of the preoperative reverse total shoulder arthroplasty patient

**DOI:** 10.1016/j.xrrt.2025.08.006

**Published:** 2025-08-16

**Authors:** Peyton L. King, Jared L. Zitnay, Mitchell S. Kirkham, Kyle B. Christy, Peter N. Chalmers, Robert Z. Tashjian, Heath B. Henninger

**Affiliations:** a Department of Orthopaedics, University of Utah, Salt Lake City, UT, USA; c School of Medicine, University of Utah, Salt Lake City, UT, USA; b Department of Biomedical Engineering, University of Utah, Salt Lake City, UT, USA; d Department of Mechanical Engineering, University of Utah, Salt Lake City, UT, USA

**Keywords:** Shoulder, Joint angle, Supine, Computed tomography, Upright, Biplane fluoroscopy

## Abstract

**Background:**

Reverse total shoulder arthroplasty is a common procedure for end-stage glenohumeral arthritis, rotator cuff disease, fracture, or failed arthroplasty. Preoperative planning software allows surgeons to assess implant placement and relies on imported bone models that come from imaging, typically a supine computed tomography (CT) scan. However, daily activities are performed upright. This study quantified the differences in scapulothoracic and glenohumeral joint angles between preoperative supine and upright imaging.

**Methods:**

Seven patients underwent preoperative supine CT and upright biplane fluoroscopy imaging. Scapulothoracic and glenohumeral joint angles in supine and upright poses were calculated using Euler angles and compared using 2-sided paired *t*-tests.

**Results:**

The scapula was 9.3 ± 12.9° more downward rotated in the upright pose than supine (*P* = .004), while the humerus was 13.2 ± 17.6° more elevated (*P* = .020). Patient-specific changes varied widely for scapulothoracic upward/downward rotation (+0.5° to −17.6°), pro/retraction (+10.4° to −22.4°), and posterior/anterior tilt (+17.4° to −9.3°), as well as glenohumeral elevation/depression (+26.7° to −4.0°), anterior/posterior plane of elevation (+19.3° to −5.6°), and internal/external axial rotation (+5.5° to −44.6°).

**Conclusion:**

Patient-specific variation in joint angles between supine and upright poses demonstrates gross changes in both magnitude and direction that exceed common thresholds in implant selection and placement. Thus, preoperative planning using supine CT may inaccurately pose bones, with consequent effects on the surgical plan, the resultant shoulder biomechanics, and clinical outcomes. Preoperative planning software should consider the influence of supine posture when orienting bones for surgical planning.

The shoulder has the largest range of motion of any joint in the human body, arising from synchronized sternoclavicular, acromioclavicular, scapulothoracic, and glenohumeral motion. Shoulders perform up to 160° of flexion, 150° of abduction, and 135° of internal/external rotation.^12^ Additionally, the glenohumeral joint is prone to degenerative rotator cuff tears and osteoarthritis that lead to pain and dysfunction. Conservative treatments include physical therapy and injections, but if unsuccessful, can necessitate joint replacement.

Anatomic total shoulder arthroplasty relies on a functional rotator cuff to maintain stability and provide motion as it does in the native shoulder.^7^ In contrast, reverse total shoulder arthroplasty (rTSA) can restore shoulder function even after massive rotator cuff tears or cuff tear arthropathy. Because of the success of rTSA, it now accounts for over 70% of shoulder replacements.^5^ As the name implies, reversing the shoulder replaces the humeral head with a polymer cup and replaces the glenoid with a metal hemisphere (ie, glenosphere). The rTSA generally shifts the glenohumeral center of rotation medially and inferiorly, using the deltoid to provide stability and primary motion of the semiconstrained implant.^20^ Implant variables such as glenosphere diameter, lateralization, and inclination, as well as the humeral neck–shaft angle, lateralization, and version can be modified to optimize patient-specific surgery.^9^^,^^13^

Preoperative planning is useful to evaluate implant selection and placement. Planning software automatically creates 3-dimensional (3D) models of patient-specific bony anatomy from computed tomography (CT) scans. Surgeons then virtually place implants to optimize parameters such as glenoid baseplate coverage, reaming depth, screw trajectories, and maximize impingement-free range of motion. The plan can additionally be used to create patient-specific drill and cutting guides to ensure accurate implant placement.^41^

While the glenohumeral joint is the focus of planning and surgery, scapulothoracic motion contributes substantially to humerothoracic motion in rTSA patients.^37^^,^^43^ Patient-specific posture and resting scapulothoracic orientation also impact postoperative range of motion and clinical outcomes.^29^^,^^31^ However, preoperative supine CT scans used for planning may not reflect upright scapulothoracic or glenohumeral posture. Scapulothoracic joint angles in supine and upright CT imaging were previously analyzed in a healthy Japanese population.^27^ In 200 shoulders, scapulae were more downward rotated (up to 12°), posteriorly tilted (up to 11°), and retracted (up to 10°) in the upright pose, even in a strict posture where subjects stood against a vertical beam during imaging. However, this study did not investigate the consequent glenohumeral changes or test natural upright postures of older pathologic adults. Posture-dependent differences of even 10° meet or exceed typical 5°-10° increments in implant selection and placement parameters that directly impact shoulder biomechanics.^11^^,^^15^^,^^18^^,^^47^

Therefore, our study quantified scapulothoracic and glenohumeral joint angles in supine (CT) and upright seated (biplane fluoroscopy) imaging of preoperative rTSA patients. We hypothesized that differences in mean joint angles between supine and upright seated poses would exceed 10° for both joints in all rotational degrees of freedom. At these levels, preoperative planning using CT-based bone alignment could yield erroneous glenohumeral relationships that influence surgical planning and resultant shoulder biomechanics and surgical outcomes if left uncorrected.

## Methods

### Participants

The study was performed under ethical approval from the University of Utah institutional review board (#71782). This retrospective analysis was limited to a convenience sample of rTSA patients (previously enrolled in a prospective study) if they had preoperative supine CT, and upright imaging with biplane fluoroscopy at both preoperative and postoperative timepoints (Table I).^36^^,^^37^ The participants were (nonconsecutively) recruited in the clinics of our treating surgeons at the university orthopedic hospital. Subjects with incomplete imaging of the spinal processes were excluded since a thorax midline plane could not reliably be defined. Patient posture was assessed using the classification of Moroder et al. and included 3 Type A (physiological posture with retracted shoulders), 3 Type B (moderate scapular drooping and hyperkyphosis), and 1 Type C (severe scapular drooping and hyperkyphosis).^29^

**Table I:** Preoperative patient demographics (N = 7, mean ± SD [range])

Sex	
Female	1
Male	6
Age (yr)	67 ± 7 [58-77]
BMI (kg/m^2^)	31 ± 3 [25-34]
Diagnosis	
Osteoarthritis	3
Cuff tear arthropathy	3
Massive rotator cuff tear	1
Posture type	
Type A	3
Type B	3
Type C	1

*SD*, standard deviation.

### Patient-specific imaging

Supine CT scans were collected per standard of care. Arm positioning was variable; all patients had their elbow at their side, but institutional protocol did not specify whether the forearm be placed with the hand on the stomach or along the thigh, supinated or pronated. The imaging field of view spanned through the thorax anterior to posterior and mediolaterally from the affected shoulder to the spine. Axially, the scans extended from above the acromion to the distal extent of the scapula (<1 mm axial slice thickness).

**Figure 1:**
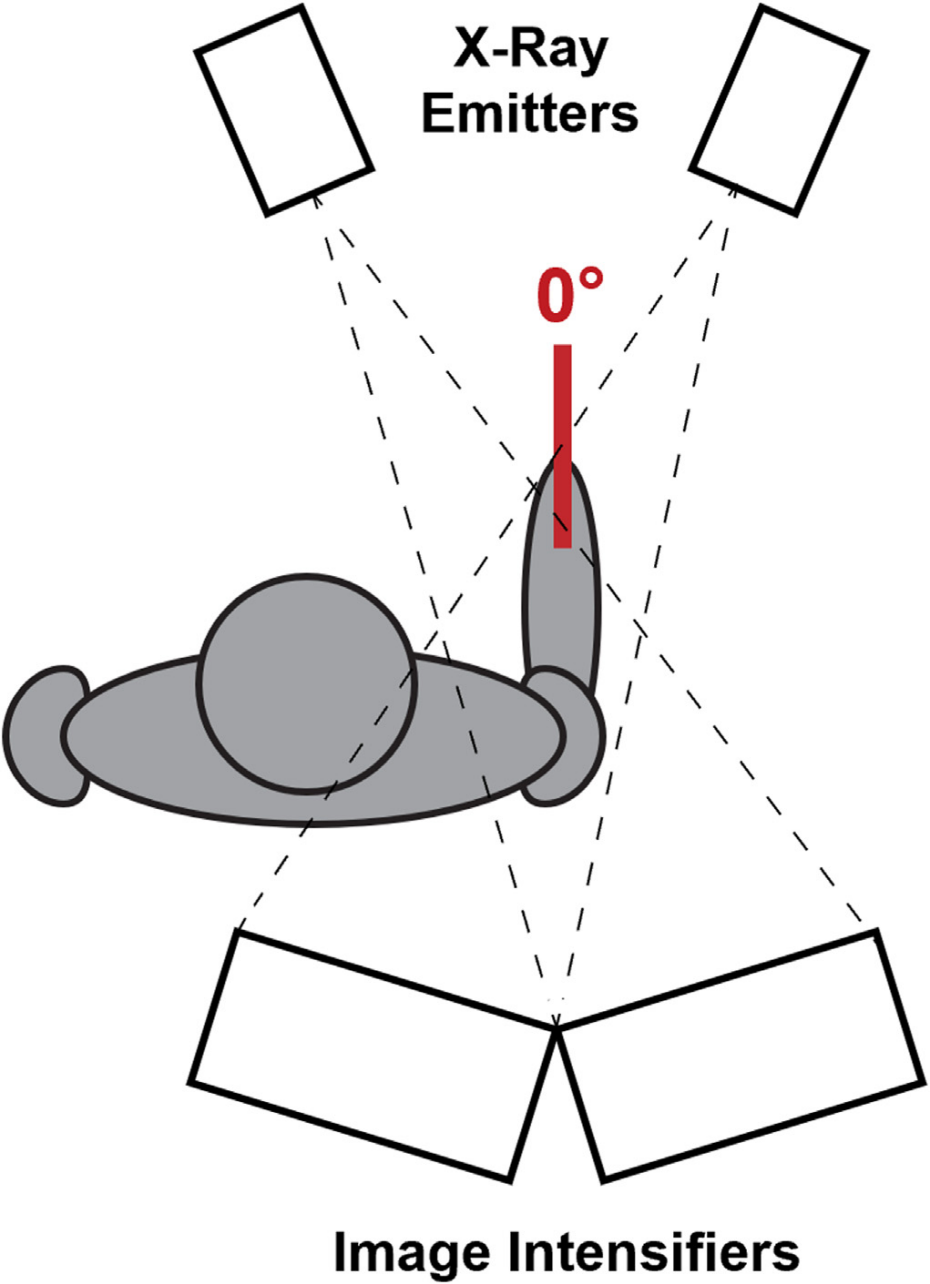
The resting neutral pose for upright imaging. The patients were seated, forearm forward, with the elbow flexed 90° and the thumb up.

For the upright seated pose, patients were instructed to sit naturally and were imaged in the resting neutral pose using a custom high-speed biplane fluoroscopy system (Fig. 1).^36^^,^^37^ Skin markers at the sternal notch, xiphoid process, and C7 spinal process were used to capture torso orientation (Vicon, Centennial, CO, USA). Optical and fluoroscopic data were spatiotemporally synchronized.

### Static pose analysis

**Figure 2:**
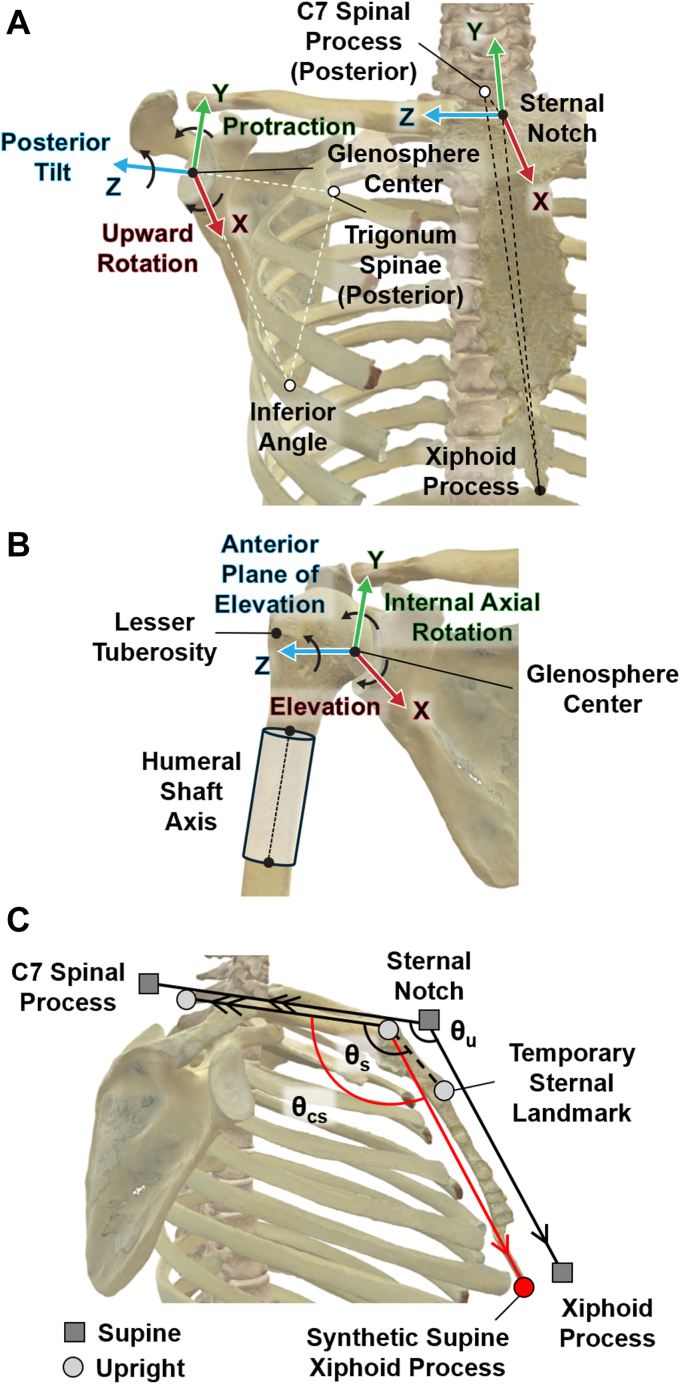
(**A**). Torso and scapular coordinate systems and rotation definitions. The torso coordinate system served as the basis so that scapula lateral (+upward) rotation, protraction (+), and posterior tilt (+) were defined relative to the thorax. Note that for more intuitive interpretation + upward did not follow the right-hand rule. (**B**) Humerus coordinate system and rotation definitions. The scapular coordinate system served as the basis so that humeral elevation (+upward), anterior plane of elevation (+), and internal axial rotation (+) were defined relative to the scapula. Note that for more intuitive interpretation + upward did not follow the right-hand rule. (**C**) Corrected supine superior-inferior axis. The synthetic xiphoid process was created in the supine system to provide consistency between supine and upright systems (θ_u_ = θ_cs_). The figures were adapted from “Shoulder Girdle” and “Axial Skeleton” by Marjan Maldoy and Ian Garcia, used under CC BY 4.0.

Patient-specific 3D models of the scapula, humerus, upper spine, and sternum were created using segmentations of CT images (Mimics 23.0; Materialise, Plymouth, MI, USA). Anatomical landmarks were identified on reconstructed 3D bone surfaces: for the torso, the sternal notch (anterior and superior at the midline), the most anterior and distal point of the sternum at the midline (not the xiphoid process, see below), and the midpoint of the C7 posterior spinous process at the midline; for the scapula the glenosphere center, inferior angle, and trigonum spinae; and for the humerus the lesser tuberosity and shaft cylinder axis (Fig. 2, *A* and *B*).^38^^,^^46^ Although preoperative imaging was analyzed, postoperative biplane fluoroscopy imaging was used to determine the location of the glenosphere implant relative to the preoperative scapula. The glenosphere center ensured a consistent and reproducible origin for both the scapula and humerus, despite anatomic variability and challenges defining landmarks on pathologic bones.^38^ The patient-specific bone models were also used for model-based, markerless tracking to find their 3D orientation in the upright pose.^37^

The torso coordinate system was guided by International Society of Biomechanics recommendations but modified to place the origin at the sternal notch.^46^ The sternum provided a common anatomic feature not influenced by posture. Due to axial CT slices ending at the distal scapula, the xiphoid process was often truncated and not visible in the supine pose. To ensure consistency between the supine and upright systems, a temporary supine superior–inferior torso axis defined by the sternal notch and distal sternum was modified using a synthetic xiphoid process landmark (Fig. 2, *C*). This ensured the calculated superior–inferior torso axis was parallel to the upright superior–inferior axis as defined by optical markers. Vectors were created from the xiphoid process to the sternal notch and from the sternal notch to the C7 spinous process in the upright (optical) system, with the angle between the vectors defined as θ_u_. The sternal notch to C7 vectors in each system were presumed parallel based on <3 mm accuracy of optical marker placement.^23^ In the supine data, the sternal notch, truncated sternum midline landmark, and C7 spinous process formed the same plane available from optical markers. The synthetic (corrected) supine xiphoid process was created within the plane so that the angle between the superior–inferior torso axis and the sternal notch to C7 vector (θ_cs_) was equal to θ_u_. The mediolateral axis was defined perpendicular to the plane formed by the sternal notch, xiphoid process, and the C7 spinal process, pointing laterally (+Z). The anteroposterior axis was defined as the cross product between the superior–inferior and mediolateral axes, pointing anteriorly (+X).

The scapula coordinate system was modified from International Society of Biomechanics (ISB) recommendations, with the origin at the glenosphere center and the inferior angle and trigonum spinae defining the scapular plane (Fig. 2, *A*).^46^ The mediolateral axis was defined from the trigonum to glenosphere center, pointing laterally (+Z). The anteroposterior axis was normal to the scapular plane, pointing anteriorly (+X). The superior–inferior axis was the cross product of the mediolateral and anteroposterior axes, pointing superiorly (+Y). The humerus coordinate system could not be defined using ISB recommendations because the clinical CT truncated the elbow epicondyles; a proximal humerus coordinate system was used instead.^38^ The origin of the humerus coordinate system was placed at the glenosphere center to avoid inaccuracies in fitting a sphere to the pathologic humeral head anatomy (Fig. 2, *B*). The humeral shaft axis was determined by selecting a region distal to the calcar where the shaft stopped tapering in a coronal view, extending distal approximately 50 mm. The cylinder fit to the cortical surface provided the superior–inferior shaft axis, pointing superiorly (+Y). The lesser tuberosity defined the anterior (+X) axis, and the cross product of the superior and anterior axes was the lateral (+Z) axis.

Scapulothoracic joint angles were calculated using a custom script (MATLAB; MathWorks, Natick, MA, USA). The ISB-recommended Y-X′-Z″ Euler sequence was used to evaluate scapular upward (lateral) and downward (medial) rotation, protraction, and tilt. The right-hand rule denoted positive rotations, but reverse sign convention was used for upward rotation for ease of interpretation (Fig. 2, *A*). Due to known issues with the Y-X′-Y″ sequence at low humeral elevations, the X-Z′-Y″ sequence was used to compute glenohumeral joint angles.^33^ The right-hand rule again denoted positive rotations, using reverse sign convention for elevation (Fig. 2, *B*).

### Statistical analysis

To compare the repeated measures of supine and upright joint angles, 2-sided paired *t*-tests were used. Statistical significance was set at *P* ≤ .05. Marginal significance was set at *P* ≤ .10 to detect differences that may be clinically relevant and warrant further analysis in future studies with larger sample sizes. Due to small sample size, the correlations between diagnoses and posture classification and joint angles were not calculated. The analyses were performed in MATLAB 2021a using the Statistics and Machine Learning Toolbox.

Interobserver and intraobserver reliability in defining anatomic landmarks on supine CT models was quantified with intraclass correlation coefficients (ICCs). Interobserver ICCs were determined using 7 torsos with 3 observers at a single time point, calculated using a single measure, absolute agreement, 2-way mixed effects model. Intraobserver ICCs were determined using 7 torsos with a single observer at 2 time points at least 1 month apart, calculated using a single measure, absolute agreement, 2-way random effects model. ICCs were calculated using Stata (IC 18.0; StataCorp, College Station, TX, USA) and evaluated as: <0.5 poor, 0.5-0.75 moderate, 0.7-0.9 good, and >0.9 excellent.^21^

Source data and code for the present analyses can be found at https://doi.org/10.5281/zenodo.17042124.

## Results

**Figure 3:**
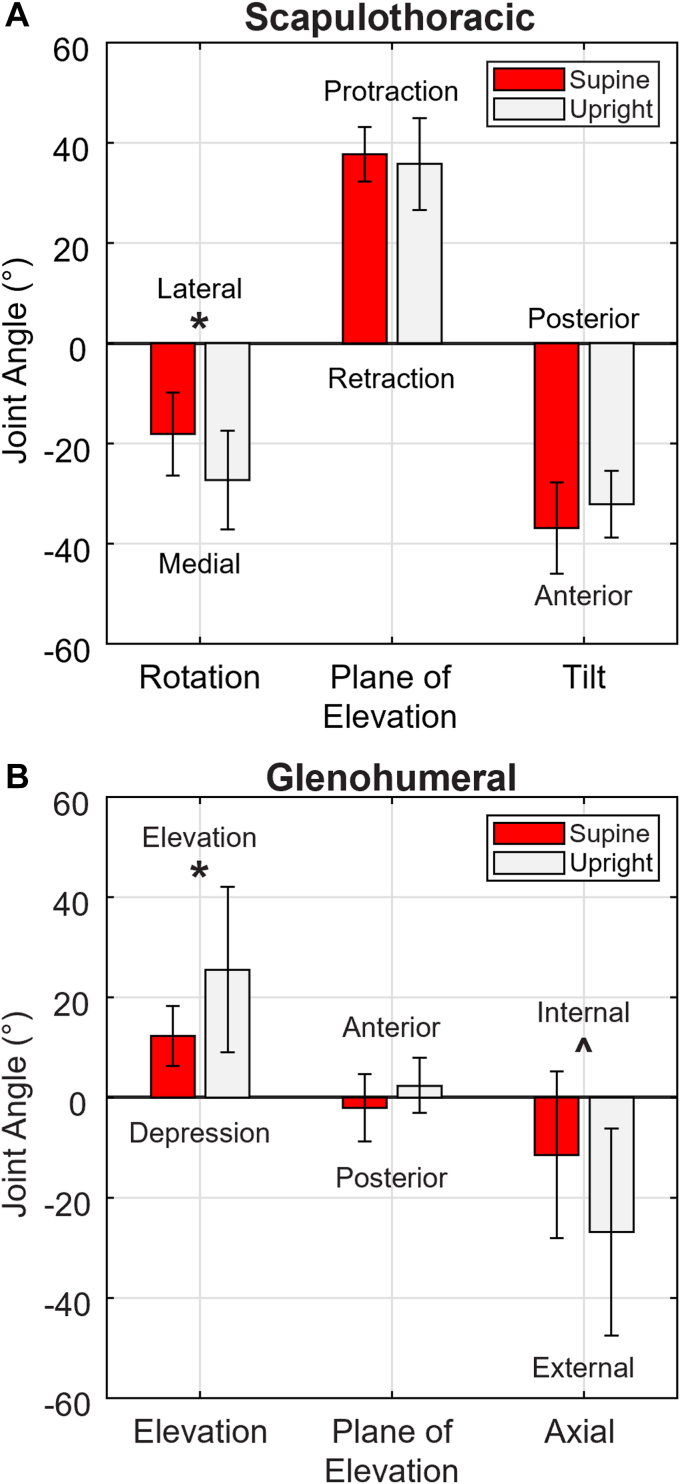
Mean scapulothoracic and glenohumeral joint angles. (**A**) Scapulothoracic joint angles in supine and upright poses. (**B)** Glenohumeral joint angles in supine and upright poses. Mean ± SD. ∗*P* ≤ .05, ˆ *P* ≤ .10. *SD*, standard deviation.

Inter-rater and intrarater ICCs for landmark selection were >0.996, and thus reliably defined. The scapula was 9.3 ± 12.9° more downward rotated in the upright pose (*P* = .004, Fig. 3, *A*). There were no differences in protraction of the scapula (*P* = .687). The upright pose trended toward more posterior scapular tilt but did not reach statistical significance (*P* = .206). The humerus was 13.2 ± 17.6° more elevated in the upright pose (*P* = .020, Fig. 3, *B*). There was no difference in plane of elevation between the upright and the supine pose (*P* = .208). The humerus was 15.4 ± 26.6° more externally rotated in the upright pose (*P* = .071).

**Figure 4:**
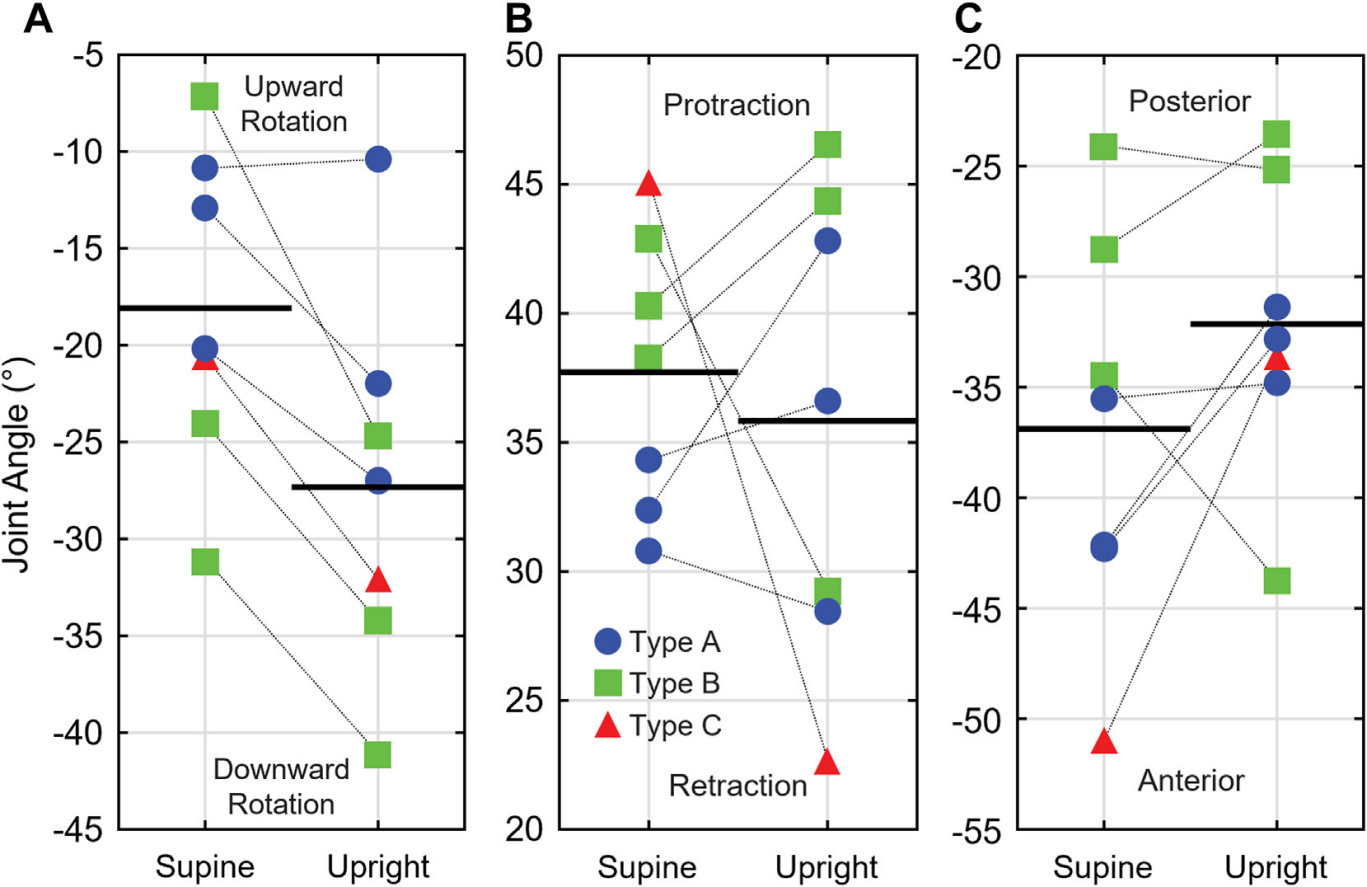
Patient-specific scapulothoracic joint angles. Scapulothoracic rotation (**A**), pro/retraction (**B**), and tilt (**C**). *Black lines* indicated sample means, where Type A posture is shown in *blue circles*, Type B posture is shown in *green squares*, and Type C posture is shown in *red triangles*.

Disparities became more apparent when analyzing patient-specific scapulothoracic changes (Fig. 4). Although 6 patients had more downward scapular rotation in the upright pose (changes ranging from −6.8° to −17.6°), 1 exhibited slightly more upward rotation (+0.5°) (Fig. 4, *A*). Four patients were more protracted in the upright pose, up to +10.4°, while 3 were more retracted, up to −22.4° (Fig. 4, *B*). Five patients demonstrated more posterior tilt in the upright pose, up to +17.4°, while 2 exhibited up to −9.3° more anterior tilt (Fig. 4, *C*).

**Figure 5:**
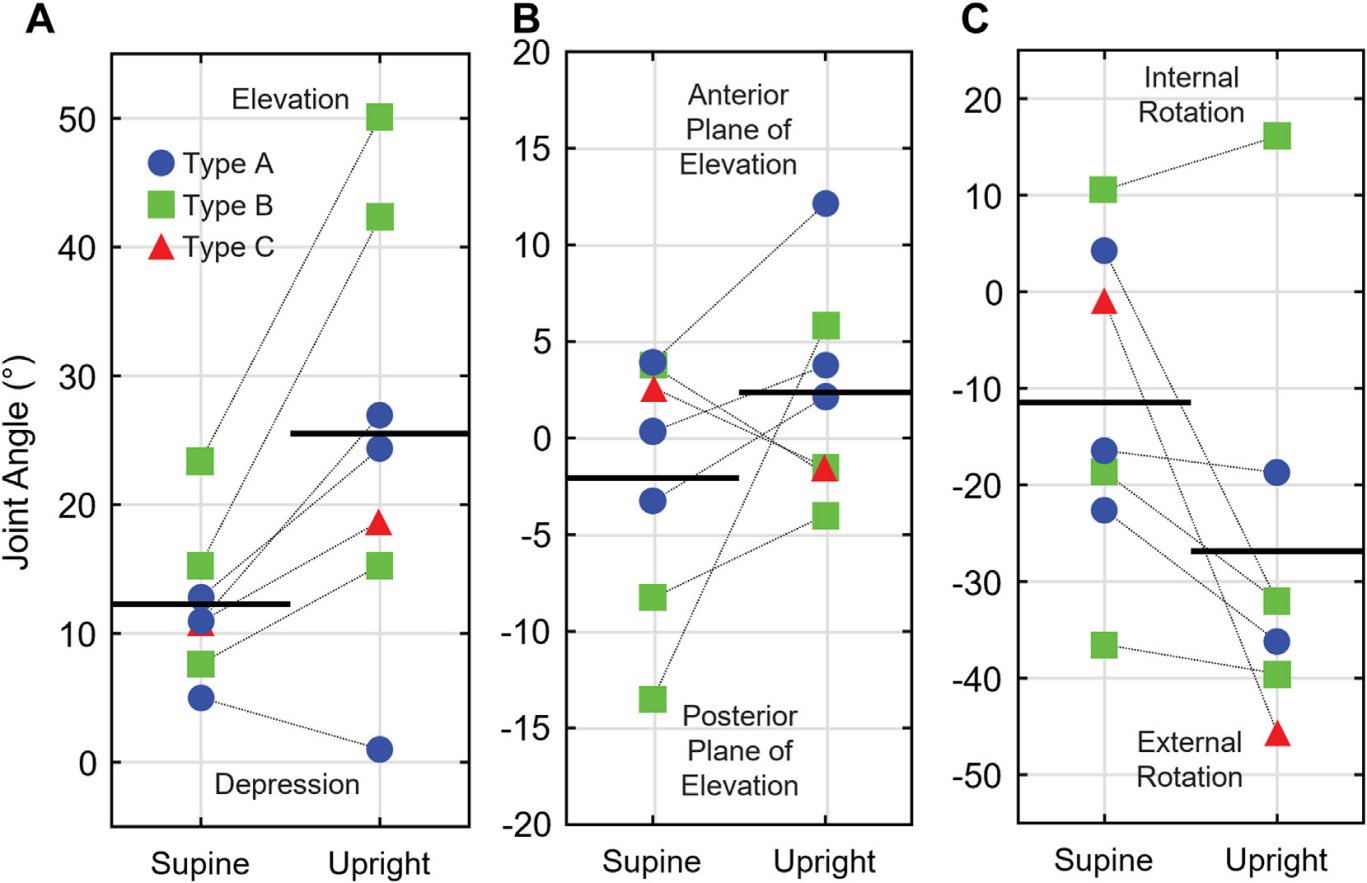
Patient-specific glenohumeral joint angles. Glenohumeral elevation (**A**), plane of elevation (**B**), and axial rotation (**C**). *Black lines* indicate sample means, where Type A posture is shown in *blue circles*, Type B posture is shown in *green squares*, and Type C posture is shown in *red triangles*.

Changes in glenohumeral joint angles among individuals also demonstrated large variability. Patient-specific elevation increased in 6 individuals (changes ranging from +7.6° to +26.7°), with 1 showing −4.0° more depression of the humerus while upright (Fig. 5, *A*). Five patients exhibited more anterior plane of elevation in the upright pose, up to +19.3°. In contrast, 2 patients exhibited up to −5.6° more posterior plane of elevation (Fig. 5, *B*). Although 6 patients experienced more external axial rotation in the upright pose (changes ranging from −3.4° to −44.6°), 1 exhibited +5.5° more internal axial rotation compared to the supine pose (Fig. 5, *C*).

## Discussion

This study compared scapulothoracic and glenohumeral joint angles in supine and upright imaging of preoperative rTSA patients. Scapulae were more downward rotated in the upright pose, and humeri were more elevated and externally rotated in the upright pose. Mean groupwise differences were near 10°, so our hypotheses were partly supported. More importantly, patient-specific differences were much larger and varied in direction, at times from 20°-45°. This wide range of individual variation is not considered in current preoperative planning when using CT data, which contradicts some of the objectives of patient-specific surgical planning, specifically the impact of the relationship of the humerus to the glenoid component.

Disparity between supine CT input data and how the patient is posed during upright daily activities could impact surgical planning and thus postoperative shoulder function and clinical outcomes. Supine CT is currently the gold standard imaging for preoperative planning. Our results suggest that changes from the supine to upright pose are patient-specific in both magnitude and direction. Therefore, a single standardized correction would not accurately reflect upright orientation for many patients, and it would still be unclear which are negatively affected. New or modified imaging techniques will need to be developed to accurately incorporate this information into the preoperative plan, but this is beyond the scope of the current study.

Scapulae were approximately −10° more downward rotated in the upright pose (up to −18°, Fig. 4, *A*), likely attributable to gravity acting on the arm and resultant muscular stabilization of the scapula.^28^ Intuitively, more downward rotated scapulae had more relatively elevated humeri in the upright pose, up to +27°. The additional differential in elevation could arise from body habitus where the curved bed of the CT scanner may crowd the arm against the torso vs. an unconstrained upright pose. Furthermore, in 1 subject, the scapula rotated nominally more upward (+0.5°) and was the only 1 to exhibit a more depressed humerus in the upright pose (−4.0°). This may be attributed to an overactive upper trapezius from compensation in avoiding preoperative pain.^42^ Regardless, these data support the coupled nature of scapulothoracic, glenohumeral, and humerothoracic resting orientations.

These relationships have biomechanical consequences. For example, glenosphere lateralization increases deltoid forces required to elevate the arm, which could limit range of motion and risk acromial fracture,^11^^,^^14^ whereas downward rotated scapulae decrease the deltoid force requirements to elevate the arm after rTSA.^18^^,^^47^ Similarly, inferior glenosphere inclination decreases superior–inferior shear forces on the glenoid component but may cause a rocking horse effect as shear forces increase and change direction during elevation in downward oriented scapulae.^18^ Glenosphere baseplate inclination is typically referenced to the local scapular anatomy, but true inclination relative to gravity depends on resting scapular orientation and the substantial increase in scapular upward rotation after rTSA.^37^^,^^43^ It is unclear if clinical outcomes are impacted by local anatomic inclination vs. inclination relative to gravity, so this phenomenon requires further investigation.

Inconsistencies in outcomes could arise from the large patient-specific variation in shoulder orientation between supine imaging and upright posture that is not captured in preoperative planning. Clinically, increased glenosphere lateralization is suggested to decrease scapular notching and increase internal/external rotation range of motion,^3^^,^^24^ but others have demonstrated no difference in functional range of motion between a lateralized and medialized glenosphere.^17^^,^^26^ Likewise, inferior inclination increases both stability and internal/external rotation of the humerus,^24^^,^^39^ while no differences were found in other studies.^16^^,^^44^ When considering current recommendations for glenosphere lateralization (4-10 mm) and inclination (neutral to 10° inferior tilt),^1^^,^^24^ the observed 18° to 27° variation in subject poses is quite large. Overlooking these factors could result in procedural decisions that negatively impact functional range of motion and exacerbate complications like glenoid loosening, scapular notching, and acromial fracture.

Scapular pro/retraction varied widely across patients but washed out when data were pooled. In contrast, humeral axial rotation more consistently shifted toward external rotation in upright poses (up to −44.6°). When supine, gravity likely pulls the scapulae into retraction as the shoulders relax, although this effect may be constrained by the CT table, pillows, or thoracic anatomy. Forearm positioning also varied, often depending on technician preference. Upright scans, on the other hand, captured patients in their more natural resting seated posture.

It is becoming understood that scapular pro/retraction plays a role in functional outcomes following rTSA. For example, during shoulder elevation, anticipatory scapular retraction facilitates greater overall range of motion.^34^ Similarly, greater scapular retraction in the resting pose has been linked to improved internal rotation performance, as it provides the humerus more clearance before impingement occurs.^36^ Surgically, increasing glenosphere retroversion has been shown to improve internal rotation and range of motion,^10^^,^^25^ although other studies report that retroversion has minimal impact, or may even reduce, internal rotation.^8^^,^^22^ These conflicting findings related to implant placement may stem from insufficient consideration of scapular pro/retraction during surgical planning and execution.

The variability in scapular pro/retraction and humeral axial rotation may also contribute to the ongoing debate surrounding optimal humeral component retroversion. Current clinical practice often bases retroversion on forearm alignment, though this approach is evolving. Studies previously recommended setting retroversion within 0°-20° to balance muscle efficiency and minimize impingement,^4^^,^^19^ yet many surgeons use 20°-30° of retroversion based on average humeral anatomy, typically without accounting for scapular position.^6^^,^^35^ To address postural variability, some have proposed matching humeral implant retroversion to the degree of scapular protraction.^29^ Clinical evidence suggests that humeral retroversion has little to no effect on functional outcomes or complication rates after rTSA,^35^^,^^45^ while others report better outcomes when retroversion aligns with the patient's native proximal humerus orientation.^32^ The lack of consensus highlights the need for more consistent and accurate preoperative imaging that incorporates scapulothoracic and glenohumeral orientation into surgical planning and execution.

No differences in scapulothoracic tilt or glenohumeral plane of elevation were observed between supine and upright poses. However, patient-specific variability remained substantial (18° and 24°, respectively). As the scapula tilts away from neutral (ie, vertical, as often assumed in planning software), glenoid inclination and version become compound measurements relative to gravity. Additionally, the alignment between the humeral shaft and neck shaft angle affects which part of the glenosphere contacts the humeral polyethylene component. This directly impacts calculations of impingement-free range of motion and may help explain discrepancies between predicted and actual functional outcomes.^2^^,^^40^ Similar to protraction, scapular tilt is posture dependent. For example, patients with severe kyphosis often exhibit increased anterior tilt, which can enhance internal rotation.^29^ Dynamic anterior tilt also aids internal rotation post-rTSA.^36^ It is worth noting that the glenohumeral plane of elevation is a sensitive measure, particularly when the arm is at rest.^33^ Among the variables studied, this measurement is likely the least relevant for preoperative planning.

This study has several limitations. First, the requirement for both the sternum and spine to be visible in CT images reduced the number of eligible participants. While the sample size was small, individual variation was large in both magnitude and direction. The use of central tendency statistics (eg, average, standard deviation) could mask discrepancies that have profound consequences on patient-specific surgery and prevent reliable corrections between supine and upright poses. Thus, future studies should consider individual variation in larger cohorts when evaluating trends that may be linked to variables like the type of kyphosis or pathology.^29^, ^30^, ^31^ The cohort included a range of pathologies that reflect the diversity typically seen in clinical practice. While trends due to pathology could not be analyzed, this variability is likely what can be expected from every-day clinical populations. Posture was classified using an existing system,^29^ but the dataset was insufficient to draw conclusions about the effects of posture. Future studies should include a larger, sex-balanced cohort with a range of preoperative diagnoses to better evaluate group trends, individual variability, and the relationship between posture and anatomical differences. The xiphoid process was not visible in supine CT scans because clinical imaging ended distal to the scapula, leading to variable truncation of the sternum and arm. To address this, we estimated a synthetic xiphoid point based on assumptions about skin-marker motion capture accuracy and known anatomical landmarks (<3 mm error^23^). Even allowing for 5° of error, the observed differences between supine and upright poses remained substantial, suggesting the findings are robust. Future clinical scans could include the entire sternum to provide a more reliable torso reference for surgical planning. Additionally, we used the glenosphere center, a postoperative landmark, as a consistent origin, though this would not be available preoperatively. Lastly, forearm positioning during the supine CT varied per standard of care, as imaging protocols do not consistently specify positioning. This variation reflects how preoperative imaging is typically performed in clinical practice and was intentionally preserved to capture real-world variability. Establishing a more consistent imaging protocol could improve data uniformity and enhance the integration of these findings into clinical workflows.

## Conclusion

Patient-specific variation in joint angles between supine and upright poses demonstrates gross changes in both magnitude and direction that exceed common thresholds in implant selection and placement. Thus, preoperative planning using supine CT may inaccurately pose bones, with consequent effects on the surgical plan, the resultant shoulder biomechanics, and clinical outcomes. Preoperative planning software should consider the influence of supine posture when orienting bones for surgical planning.

## Disclaimers

Funding: This study was supported by the 10.13039/100000069National Institute of Arthritis and Musculoskeletal and Skin Diseases of the 10.13039/100000002National Institutes of Health under award numbers R01 and R56 AR067196, and a Shared Instrumentation Grant S10 OD021644. The research content herein is solely the responsibility of the authors and does not necessarily represent the official views or contributions of the sponsors.

Conflicts of interest: Robert Z. Tashjian is a paid consultant for Zimmer Biomet, Enovis, and Stryker; has stock in Conextions and Genesis; receives intellectual property royalties from Zimmer Biomet, Stryker, and Shoulder Innovations; receives publishing royalties from Springer and the *Journal of Bone and Joint Surgery*; and serves on the editorial board of the *Journal of Bone and Joint Surgery*. Peter N. Chalmers is a paid consultant for DePuy, Smith + Nephew, Responsive Arthroscopy, and Exactech; receives intellectual property royalties from DePuy, Responsive Arthroscopy, and Exactech; has stock in TitinKM Biomedical; receives research support from DePuy, Smith + Nephew, and the National Institutes of Health; and serves on the editorial board of the *Journal of Shoulder and Elbow Surgery*. Heath B. Henninger receives research support from Smith + Nephew, Stryker, and the National Institutes of Health. The other authors, their immediate families, and any research foundation with which they are affiliated have not received any financial payments or other benefits from any commercial entity related to the subject of this article.

Given his role as Editor in Chief, Dr Peter N. Chalmers had no involvement in the peer-review of this article and has no access to information regarding its peer-review. Full responsibility for the editorial process for this article was delegated to Dr John W. Sperling.

## References

[1] Berhouet J., Garaud P., Favard L. (2014). Evaluation of the role of glenosphere design and humeral component retroversion in avoiding scapular notching during reverse shoulder arthroplasty. J Shoulder Elbow Surg.

[2] Berhouet J., Samargandi R., Favard L., Turbillon C., Jacquot A., Gauci M.O. (2023). The real post-operative range of motion differs from the virtual pre-operative planned range of motion in reverse shoulder arthroplasty. J Pers Med.

[3] Berton A., Gulotta L.V., Longo U.G., De Salvatore S., Piergentili I., Bandini B. (2021). Medialized versus lateralized center of rotation in reverse total shoulder arthroplasty: a systematic review and meta-analysis. J Clin Med.

[4] Berton A., Gulotta L.V., Petrillo S., Florio P., Longo U.G., Denaro V. (2015). The effect of humeral version on teres minor muscle moment arm, length, and impingement in reverse shoulder arthroplasty during activities of daily living. J Shoulder Elbow Surg.

[5] Best M.J., Aziz K.T., Wilckens J.H., McFarland E.G., Srikumaran U. (2021). Increasing incidence of primary reverse and anatomic total shoulder arthroplasty in the United States. J Shoulder Elbow Surg.

[6] Boileau P., Walch G. (1997). The three-dimensional geometry of the proximal humerus. Implications for surgical technique and prosthetic design. J Bone Joint Surg Br.

[7] Boudreau S., Boudreau E.D., Higgins L.D., Wilcox R.B. (2007). Rehabilitation following reverse total shoulder arthroplasty. J Orthop Sports Phys Ther.

[8] Budge M., Lewis G., Vanname J. (2021). The effect of glenoid version on internal and external rotation in reverse total shoulder arthroplasty. Semin Arthroplasty JSES.

[9] Elwell J., Athwal G., Willing R. (2021). Maximizing range of motion of reverse total shoulder arthroplasty using design optimization techniques. J Biomech.

[10] Galasso L.A., Clinger B.N., Werner B.C., Denard P.J., Shoulder Arthroplasty Research C (2025). Increased glenoid baseplate retroversion improves internal rotation following reverse shoulder arthroplasty. JSES Int.

[11] Giles J.W., Langohr G.D., Johnson J.A., Athwal G.S. (2015). Implant design variations in reverse total shoulder arthroplasty influence the required deltoid force and resultant joint load. Clin Orthop Relat Res.

[12] Gill T.K., Shanahan E.M., Tucker G.R., Buchbinder R., Hill C.L. (2020). Shoulder range of movement in the general population: age and gender stratified normative data using a community-based cohort. BMC Musculoskelet Disord.

[13] Glenday J.D. (2018).

[14] Henninger H.B., Barg A., Anderson A.E., Bachus K.N., Burks R.T., Tashjian R.Z. (2012). Effect of lateral offset center of rotation in reverse total shoulder arthroplasty: a biomechanical study. J Shoulder Elbow Surg.

[15] Henninger H.B., King F.K., Tashjian R.Z., Burks R.T. (2014). Biomechanical comparison of reverse total shoulder arthroplasty systems in soft tissue-constrained shoulders. J Shoulder Elbow Surg.

[16] Kempton L.B., Balasubramaniam M., Ankerson E., Wiater J.M. (2011). A radiographic analysis of the effects of glenosphere position on scapular notching following reverse total shoulder arthroplasty. J Shoulder Elbow Surg.

[17] King J.J., Hones K.M., Wright T.W., Roche C., Zuckerman J.D., Flurin P.H. (2023). Does isolated glenosphere lateralization affect outcomes in reverse shoulder arthroplasty?. Orthop Traumatol Surg Res.

[18] Knighton T.W., Chalmers P.N., Sulkar H.J., Aliaj K., Tashjian R.Z., Henninger H.B. (2022). Reverse total shoulder glenoid component inclination affects glenohumeral kinetics during abduction: a cadaveric study. J Shoulder Elbow Surg.

[19] Kontaxis A., Chen X., Berhouet J., Choi D., Wright T., Dines D.M. (2017). Humeral version in reverse shoulder arthroplasty affects impingement in activities of daily living. J Shoulder Elbow Surg.

[20] Kontaxis A., Johnson G.R. (2009). The biomechanics of reverse anatomy shoulder replacement--a modelling study. Clin Biomech.

[21] Koo T.K., Li M.Y. (2016). A guideline of selecting and reporting intraclass correlation coefficients for reliability research. J Chiropr Med.

[22] Lansdown D., Cheung E.C., Xiao W., Lee A., Zhang A.L., Feeley B.T. (2020). Do preoperative and postoperative glenoid retroversion influence outcomes after reverse total shoulder arthroplasty?. J Shoulder Elb Arthroplast.

[23] Lewis J., Green A., Reichard Z., Wright C. (2002). Scapular position: the validity of skin surface palpation. Man Ther.

[24] Li X., Knutson Z., Choi D., Lobatto D., Lipman J., Craig E.V. (2013). Effects of glenosphere positioning on impingement-free internal and external rotation after reverse total shoulder arthroplasty. J Shoulder Elbow Surg.

[25] Mahylis J.M., Friedman R.J., Elwell J., Kasto J., Roche C., Muh S.J. (2024). Anatomic vs. reverse total shoulder arthroplasty with glenoid retroversion of at least 15 degrees in rotator cuff intact patients: a comparison of short-term results. Semin Arthroplasty JSES.

[26] Marigi E.M., Esper R.N., Larson D.R., Morrey M.E., Sanchez-Sotelo J. (2024). Effect of glenosphere diameter and lateralization in primary reverse shoulder arthroplasty: a randomized clinical trial. Semin Arthroplasty JSES.

[27] Matsumura N., Yamada Y., Oki S., Yoshida Y., Yokoyama Y., Yamada M. (2020). Three-dimensional alignment changes of the shoulder girdle between the supine and standing positions. J Orthop Surg Res.

[28] Micoogullari M., Uygur S.F., Yosmaoglu H.B. (2023). Effect of scapular stabilizer muscles strength on scapular position. Sports Health.

[29] Moroder P., Akgun D., Plachel F., Baur A.D.J., Siegert P. (2020). The influence of posture and scapulothoracic orientation on the choice of humeral component retrotorsion in reverse total shoulder arthroplasty. J Shoulder Elbow Surg.

[30] Moroder P., Siegert P., Coifman I., Ruttershoff K., Spagna G., Scaini A. (2024). Scapulothoracic orientation has a significant influence on the clinical outcome after reverse total shoulder arthroplasty. J Shoulder Elbow Surg.

[31] Moroder P., Urvoy M., Raiss P., Werthel J.D., Akgun D., Chaoui J. (2022). Patient posture affects simulated ROM in reverse total shoulder arthroplasty: a modeling study using preoperative planning software. Clin Orthop Relat Res.

[32] Oh J.H., Sharma N., Rhee S.M., Park J.H. (2020). Do individualized humeral retroversion and subscapularis repair affect the clinical outcomes of reverse total shoulder arthroplasty?. J Shoulder Elbow Surg.

[33] Phadke V., Braman J.P., LaPrade R.F., Ludewig P.M. (2011). Comparison of glenohumeral motion using different rotation sequences. J Biomech.

[34] Reina M., Fiumana G., Mantovani M., D'Antonio L., Porcellini G. (2023). Scapulohumeral rhythm in shoulders with reverse shoulder arthroplasty measured with a new portable three-dimensional scapular kinematics assessment system. J Shoulder Elbow Surg.

[35] Rhee Y.G., Cho N.S., Moon S.C. (2015). Effects of humeral component retroversion on functional outcomes in reverse total shoulder arthroplasty for cuff tear arthropathy. J Shoulder Elbow Surg.

[36] Sulkar H.J., Aliaj K., Tashjian R.Z., Chalmers P.N., Foreman K.B., Henninger H.B. (2023). High and low performers in internal rotation after reverse total shoulder arthroplasty: a biplane fluoroscopic study. J Shoulder Elbow Surg.

[37] Sulkar H.J., Aliaj K., Tashjian R.Z., Chalmers P.N., Foreman K.B., Henninger H.B. (2022). Reverse total shoulder arthroplasty alters humerothoracic, scapulothoracic, and glenohumeral motion during weighted scaption. Clin Orthop Relat Res.

[38] Sulkar H.J., Zitnay J.L., Aliaj K., Henninger H.B. (2021). Proximal humeral coordinate systems can predict humerothoracic and glenohumeral kinematics of a full bone system. Gait Posture.

[39] Tashjian R.Z., Martin B.I., Ricketts C.A., Henninger H.B., Granger E.K., Chalmers P.N. (2018). Superior baseplate inclination is associated with instability after reverse total shoulder arthroplasty. Clin Orthop Relat Res.

[40] Thomas L.G., Chalmers P.N., Henninger H.B., Davis E.W., Tashjian R.Z. (2024). Preoperative planning software does not accurately predict range of motion in reverse total shoulder arthroplasty. J Am Acad Orthop Surg.

[41] Throckmorton T.W., Gulotta L.V., Bonnarens F.O., Wright S.A., Hartzell J.L., Rozzi W.B. (2015). Patient-specific targeting guides compared with traditional instrumentation for glenoid component placement in shoulder arthroplasty: a multi-surgeon study in 70 arthritic cadaver specimens. J Shoulder Elbow Surg.

[42] Turgut E., Duzgun I., Baltaci G. (2016). Effect of trapezius muscle strength on three-dimensional scapular kinematics. J Phys Ther Sci.

[43] Walker D., Matsuki K., Struk A.M., Wright T.W., Banks S.A. (2015). Scapulohumeral rhythm in shoulders with reverse shoulder arthroplasty. J Shoulder Elbow Surg.

[44] Werner B.C., Griffin J.W., Lederman E., Gobezie R., Denard P.J. (2021). Glenosphere inclination and clinical outcomes after reverse shoulder arthroplasty. Semin Arthroplasty JSES.

[45] Wiater J.M., Lee J.Y.J., Shields E.J.W., Childers K., Dery L., Koueiter D. (2024). Humeral component version has no effect on outcomes following reverse total shoulder arthroplasty: a prospective, Double-blinded, randomized controlled trial. J Bone Joint Surg Am.

[46] Wu G., van der Helm F.C., Veeger H.E., Makhsous M., Van Roy P., Anglin C. (2005). ISB recommendation on definitions of joint coordinate systems of various joints for the reporting of human joint motion--Part II: shoulder, elbow, wrist and hand. J Biomech.

[47] Zitnay J.L., Tashjian R.Z., Walch G., Chalmers P.N., Joyce C.D., Henninger H.B. (2024). Inlay vs. onlay humeral components in reverse total shoulder arthroplasty: a biorobotic shoulder simulator study. J Shoulder Elbow Surg.

